# Analysis growth development of 153 disorders of sex development

**DOI:** 10.1186/1687-9856-2013-S1-O27

**Published:** 2013-10-03

**Authors:** Wu Di, Gong Chunxiu, Qin Miao

**Affiliations:** 1 Beijing Children’s Hospital Affiliated to Capital Medical University, Beijing, China

## Objective

To analyze clinical characteristics and growth development of 153 disorders of sex development (DSD) and non-CAH patients.

## Method

To collect DSD patients’ clinical data, including age, gender and family history. To definite categories and assess height, weight and measure penis length and testis volume and describe the deformation. Determine the levels of sex hormones. HCG test, B ultrasonic and chromosomes were performed.

## Result

Chief complain of all these patients to see doctors were abnormal external genitalia. I53 DSD patients include social sex 128 male and 25 female from 42 days to16y10m (4.59±4.25y). There were 121 (90.3%) 46XYDSD. 39 cases(32.2%) were diagnosed as hypospadias combined with micropenis, 19(15.7%) were micropenis with testis abnormality and 18 cases(14.9%) were simple micropenis. 13(10.7%) were testis abnormality, 9(7.4%) were hypospadias, 1 (0.8%) were hypospadias combined with testis abnormality, micropenis combined with hypospadias and testis abnormality were 14(11.6%). 15 (9.8%) cases had DSD family histories and 19 (12.4%) patients’ mother had taken progesterone when early pregnant stage threatened abortion. With face/limbs malformation or mental problems were 17 (11.1%) . Most of DSD were shorter than the normal population, 16 (10.4%) was <-2SDS, 115(75.2%)<1SDS. The proportion of height shorter than 25 percentile and 50 percentile was more than normal population, P=0.039 and 0.056 respectively. There were 40 cases with testis abnormality DSD, whose height were shorter than normal population (P=0.041and 0.015). 130/153 DSD were performed HCG test, height>P50 was 60.3% among 78 who had normal testorone respond, while only 18.2% height>P50 among 33 cases with abnormal testorone respond (P=0.000).

## Conclusion

This non-CAH DSD included 46XYDSD(121cases), 46XXDSD(3cases) and chromosome DSD(10cases). The most number of 46XYDSD was hypospadias combined with micropenis, the second was micropenis. Some patients had DSD family histories and some patients whose mother had been threatened abortion and progesterone exposure duration pregnancy. DSD may have malformation of face/ limbs or internal organs or mental problems. Patients with DSD may have both disorders of sex development and short statures. Most of DSD were shorter than normal population and testicular development was related to short statures. So when we evaluated DSD, not only should pay attention to sex development but also to short statures.

